# microRNA-21-5p from M2 macrophage-derived extracellular vesicles promotes the differentiation and activity of pancreatic cancer stem cells by mediating KLF3

**DOI:** 10.1007/s10565-021-09597-x

**Published:** 2021-03-17

**Authors:** Jian Chang, Hanjun Li, Zhongchao Zhu, Pei Mei, Weimin Hu, Xingcheng Xiong, Jing Tao

**Affiliations:** Department of Pancreatic Surgery, Renmin Hospital of Wuhan University, 238 Jiefang Road, Wuhan, 430060 Hubei China

**Keywords:** Pancreatic cancer, Stemness, M2 macrophages, Extracellular vesicles, MicroRNA-21a-5p, Krüppel-like factor 3, Differentiation

## Abstract

**Aim:**

Given the fact that tumor-associated macrophage-derived extracellular vesicles (EVs) are attributable to tumor aggressiveness, this research intends to decode the mechanism of M2 macrophage-derived EVs in the differentiation and activities of pancreatic cancer (PaCa) stem cells via delivering microRNA (miR)-21-5p.

**Methods:**

Polarized M2 macrophages were induced, from which EVs were collected and identified. miR-21-5p expression in M2 macrophage-derived EVs was tested. After cell sorting, CD24^+^CD44^+^EpCAM^+^ stem cells were co-cultured with M2 macrophages, in which miR-21-5p was upregulated or downregulated. The effects of M2 macrophage-derived EVs and miR-21-5p on Nanog/octamer-binding transcription factor 4 (Oct4) expression, sphere formation, colony formation, invasion and migration capacities, apoptosis, and in vivo tumorigenic ability were examined. Krüppel-like factor 3 (KLF3) expression and its interaction with miR-21-5p were determined.

**Results:**

M2 macrophage-derived EVs promoted PaCa stem cell differentiation and activities. miR-21a-5p was upregulated in M2 macrophage-derived EVs. miR-21a-5p downregulation in M2 macrophage-derived EVs inhibited Nanog/Oct4 expression and impaired sphere-forming, colony-forming, invasion, migration, and anti-apoptosis abilities of PaCa stem cells in vitro and tumorigenic ability in vivo. miR-21-5p targeted KLF3 to mediate the differentiation and activities of PaCa stem cells, and KLF3 was downregulated in PaCa stem cells.

**Conclusion:**

This work explains that M2 macrophage-derived exosomal miR-21a-5p stimulates differentiation and activity of PaCa stem cells via targeting KLF3, paving a novel way for attenuating PaCa stemness.

**Graphical abstract:**

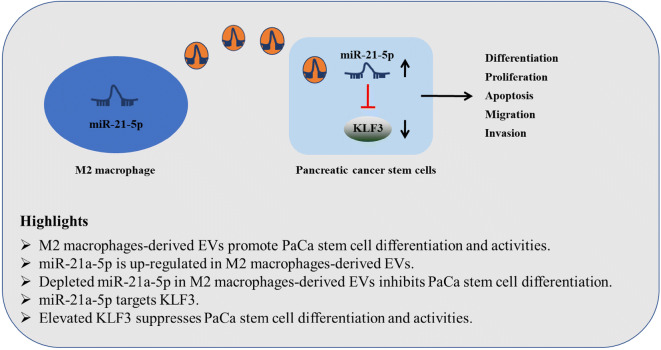

## Introduction

Pancreatic cancer (PaCa) is registered as the 4th inducement for cancer mortality and often diagnosed at an advanced or metastatic stage (Iglesia et al. 2020). Over 85% PaCa cases are presented in the form of pancreatic ductal adenocarcinoma (PDAC) (Hsieh et al. 2020). Through received with surgical treatment or neoadjuvant/adjuvant chemotherapy, the prognosis of PaCa patients is far below the expectancy (Wang et al. 2020b). Predominantly, resistance to chemotherapy is listed for the obstacle for treatment of PaCa patients (Kim et al. 2020). Indicatively, cancer stem cells and epithelial-mesenchymal transition (EMT) are the involved actors to promote PaCa cell metastasis and drug resistance (Wang et al. 2020c). Given the invasive properties of PaCa cancer stem cells, PaCa is probably managed by overcoming the stemness of PaCa cells.

M2 macrophages, the abundant immunosuppressive cell types in the tumor microenvironment of PDAC, offer the niche for parental PDAC cells to migrate, invade, and self-renew (Chandrakesan et al. 2020). M2 macrophages threaten the survival of PaCa patients and impair the efficacy of chemotherapy (Liu et al. 2020b). Moreover, M2 macrophage infiltration takes responsibility for the unexpected survival of PDAC patients (Hu et al. 2020). Exosomes serve to incite the progressive development of tumors in the microenvironment via transporting genes (Su et al. 2016). M2 macrophage-derived exosomes are the activator for PaCa cell progression and tumor growth (Yin et al. 2020). Impressively, M2 macrophage-derived exosomes, along with exosomal microRNA (miR)-501-3p are both the promoter of PDAC cell invasion, migration, metastasis, and tumor formation (Yin et al. 2019). In PDAC, miR-21-5p exerts as an oncogenic miRNA and the specified miRNA in discriminating pancreatic neoplasia (Gilles et al. 2019). Raised miR-21-5p expression is inevitably connected to overall survival of PDAC patients and indicated with prognostic significance (Karasek et al. 2018). Concretely, M2 macrophage-derived exosomes contain upregulated miR-21-5p, which may involve in the regulation of migratory and invasive actions of colorectal cancer (CRC) cells (Lan et al. 2019). Krüppel-like factors (KLFs) are the transcriptional factors that function critically in PaCa. As the subset of KLFs, KLF3 has been revealed to be a target gene of miR-21 (Zhai et al. 2019). Interestingly, it is indicated to connect with stem cell-like properties in esophageal squamous cell carcinoma (Liu et al. 2020a). Furthermore, KLF3 has the potential to improve prognostic prediction of prostate cancer (Meng et al. 2020). Consulted from these researches, this work is put forward with the hypothesis that M2 macrophage-derived extracellular vesicles (EVs) elevate miR-21-5p which targets KLF3 to affect PaCa stem cell differentiation and activity.

## Methods and materials

### Ethics statement

All animal experiments were approved by the Animal Protection and Use Committee of Renmin Hospital of Wuhan University.

### Cell culture

Human PaCa cell lines (PC-3, Capan-1, AsPC-1 and PANC-1), normal pancreatic cells HPC-Y5, and human monocytes THP-1 were available from ATCC (VA, USA). Cultivation for PC-3, Capan-1, AsPC-1, and PANC-1 cell lines was conducted in Dulbecco’s modified Eagle’s medium (DMEM) of 10% fetal bovine serum (FBS) and 1% penicillin-streptomycin while that for THP-1 cells in Roswell Park Memorial Institute-1640 medium (Gibco, CA, USA) of 10% FBS and 1% penicillin-streptomycin. FBS and penicillin-streptomycin were both from Gibco. THP-1 cells of 90% confluence were detached by 0.25% trypsin (Solarbio Science & Technology Co., Ltd., Beijing, China) and passaged. Then, 100 ng/mL phorbol myristic acetate (Sigma-Aldrich, CA, USA) was utilized in 24-h culture of THP-1 cells and 20 ng/mL interleukin (IL)-4 and IL-13 (Peprotech, NJ, USA) in 48-h induction of M2 macrophage polarization (Chen et al. 2019; Snodgrass et al. 2020; Becerra-Diaz et al. 2021). F4/80, CD86, and CD206 were determined by flow cytometry (Zhu et al. 2020) whereas Arginase-1 (ARG-1) and IL-10 (M2 macrophage-related cytokines) by reverse transcription quantitative polymerase chain reaction (RT-qPCR) (Guan et al. 2020).

### CD24^+^CD44^+^EpCAM^+^ cell sorting

The PANC-1 and AsPC-1 cell suspensions (1 × 10^7^ cells/mL, 100 μL) were incubated with 20 μL CD24, CD44, and EpCAM antibodies (Abcam, MA, USA) on ice. Then, cells were centrifuged (400 g, 4 °C) with phosphate-buffered saline (PBS), and sorted with 500 μL PBS on a flow cytometer (FACS AriaII, BD Biosciences, NJ, USA). CD24^+^CD44^+^EpCAM^+^ cells isolated from PANC-1 and AsPC-1 cells were PaCa stem cells (P-CSCs and A-CSCs). The P-CSCs and A-CSCs were cultivated in serum-free DMEM/F-12 of 1% N_2_, 2% B27 (both from Invitrogen, CA, USA), 20 ng/mL fibroblast growth factor, and 100 ng/mL epidermal growth factor (both from Sigma-Aldrich) (Tataranni et al. 2019).

### Isolation and identification of EVs

M2 macrophages at 80–90% confluence were cultured with exosome-depleted serum (A27208-01, Invitrogen) for 24 h, and EVs were separated by ultracentrifugation. Then, the supernatant was treated with differential centrifugation and then resuspended in 100-μL sterile PBS (Fang et al. 2018).

The EVs were diluted at 1:10, stained with 2% uranyl acetate, and transferred into a coated copper mesh for morphological observation under a transmission electron microscope (TEM, JEOL, Japan). The diluted EVs at 1:50 were subjected to size distribution analysis by the NanoSight N300 (Malvern Instruments, Malvern, UK), followed by nanoparticle tracking analysis (NTA). Western blot assay was indicated to determine CD63, CD9, and CD81 expression in EVs (Chen et al. 2020).

### Cell transfection

M2 macrophages were transfected with upregulated or downregulated miR-21-5p lentivirus, along with their negative control (NC) lentivirus. P-CSCs and A-CSCs were transfected with upregulated KLF3 lentivirus (LV-KLF3) and KLF3 LV-NC. The lentiviruses were all from Genechem (Shanghai, China). Briefly, cells were seeded in 6-well plates a day before transfection and transfected with lentivirus and 10 μg/mL Polybrene when reaching 60% confluence. Reacted for 48 h, cells were cultured with 5 μg/mL puromycin for 1 week to screen the stably transfected cells.

### Uptake of EVs

FITC-miR-21-5p (green) was electroporated into M2 macrophages, EVs were extracted, added with Dil (red), and incubated PaCa cells for 48 h. The co-localization of Dil and FITC was observed in recipient PaCa cells.

### RT-qPCR

Total RNA from EVs and cells was extracted by Trizol (Invitrogen), after which reverse transcription was completed on a PrimeScript^TM^ RT reagent Kit (Takara, Shiga, Japan) and qPCR on the SYBR Premix Ex TaqTM II (Takara) and ABI7500 (ABI, USA) instrument. Glyceraldehyde-3-phosphate dehydrogenase (GAPDH) referred to the internal control. 2^-ΔΔCt^ method was employed to data evaluation. Primer sequences were exhibited in Table 1.

**Table 1:** Primer sequences

Genes	Primer sequences (5’-3’)
miR-21-5p	F: TAGCTTATCAGACTGATGTTGA
	R: AGTGCGTGTCGTGG
KLF3	F: TGTCTCAGTGTCATACCCATCT
	R: CCTTCTGGGGTCTGAAAGAACTT
GAPDH	F: GGTCGGAGTCAACGGATTTG
	R: ATGAGCCCCAGCCTTCTCCAT
ARG-1	F: CATATCTGCCAAAGACATCGTG
	R: GACATCAAAGCTCAGGTGAATC
IL-10	F: ACCAAGACCCAGACATCA
	R: ATTCTTCACCTGCTCCAC
Nanog	F: GTCCCGGTCAAGAAACAGAA
	R: TGCGTCACACCATTGCTATT
Oct4	F: GTGGAGAGCAACTCCGATG
	R:TGCTCCAGCTTCTCCTTCTC

Note: *F* forward, *R* reverse, *miR-21-5p* microRNA-21-5p, *KLF3* Krüppel-like factor 3, *GAPDH* glyceraldehyde-3-phosphate dehydrogenase, *ARG-1* Arginase-1, *IL-10* interleukin-10, *Oct4* octamer-binding transcription factor 4

### Western blot assay

Cells or EVs cultured in 6-well plates were added with 100 μL radio-immunoprecipitation assay lysis buffer (Beyotime) and 1 μL phenylmethanesulfonyl fluoride, followed by reaction on ice for 30 min and centrifugation at 13000 rpm (30 min, 4 °C). After protein determination by Bradford method (Beyotime), the protein was separated by 12% sodium dodecyl sulphate polyacrylamide gel electrophoresis and transferred to a polyvinylidene fluoride membrane. The protein membrane, sealed in 5% skim milk, was tested with the primary antibodies CD63 (1:1000), CD9 (1:1000), CD81 (1:1000), KLF3 (1:1000, 39 kDa, all from Abcam), and with horseradish peroxidase (HRP)-labeled secondary antibody. GAPDH was the internal control. The membrane was reacted with enhanced chemiluminescence (Millipore, MA, USA; A and B luminescent solutions were mixed at 1:1). The membrane was exposed for 1–5 min and photographed.

### Flow cytometry

The induced M2 macrophages were probed with F4/80 (1:500), CD86 (1:500), CD206 (1:200), or isotype immunoglobulin G 2b (1:300, all from Abcam). Then, the M2 macrophages (1 × 10^6^ cells/mL) were detected on a flow cytometer (FACSCalibur, BD Biosciences) for measuring the proportion of CD86^+^ and CD206^+^ cells. M2 macrophages were F4/80^+^ and CD206^+^. Cell apoptosis was tested by Annexin V-fluorescein isothiocyanate (FITC)/propidium iodide (PI) apoptosis detection kit (Invitrogen). PANC-1 and AsPC-1 cells, and P-CSCs and A-CSCs were added with dimethyl sulfoxide (10 μmol/mL) or anti-tumor drug perifosine (10 μmol/mL) (Zhou et al. 2020). Treated for 48 h, cells were fixed in 70% ethanol, adjusted to 1 × 10^6^ cells/mL with 1× Binding Buffer, and stained by 5 μL Annexin V-FITC and 1 μL PI (Invitrogen). The flow cytometer (FACSCalibur) was adopted to apoptosis detection.

### Tumor sphere formation assay

P-CSCs and A-CSCs were resuspended in stem cell complete medium to reach 1 × 10^4^ cells/mL. Then, the cell suspensions were seeded on six-well plates (Corning, NY, USA) with the medium refreshed every 3 days. The number of cell spheres was counted under a microscope (Nikon, Tokyo, Japan) after 2 weeks. Sphere formation rate = number of spheres/number of cells (1000 cells per well in the 6-well plate) × 100% (Cao et al. 2020).

### Colony formation assay

The completely dissolved matrigel (Corning) was diluted with complete stem cell culture medium at 1:1. A cell suspension (1 × 10^5^ cells/mL, 100 μL) was added with 900 μL matrigel and spread on a 6-well plate. When the matrigel was completely solidified, the stem cell complete medium (1 mL) was added, with the medium replaced every 2–3 days. At 2 weeks post incubation, cells were fixed with 4% paraformaldehyde, added with 0.1% crystal violet, and observed by a microscope. ImageJ software was applied to colony analysis.

### Transwell assay

The Transwell (Corning) chamber with 8-μm microporous polycarbonate membrane was placed in a 24-well plate. P-CSCs and A-CSCs were resuspended in fresh serum-free DMEM/F-12 medium to attain 1 × 10^6^ cells/mL, of which 100 μL was added into the upper chamber. The lower chamber was joined with 800 μL DMEM/F-12 medium of 15% FBS (Gibco). The cells in the upper chamber were scraped off with a cotton swab, and those migrated to the bottom of the membrane were fixed with 4% formaldehyde, after which 0.5% crystal violet solution staining was conducted. The migrated cells were photographed and counted under the microscope. In the invasion assay, a matrigel (Corning) was diluted with pre-cooled serum-free DMEM/F-12 medium to 1 mg/mL, of which 100 μL was added to the upper chamber to coagulate. The other steps were the same as the migration assay.

### Immunohistochemistry

Immunohistochemistry was exploited to detect Nanog and octamer-binding transcription factor 4 (Oct4) in the subcutaneously xenografted tumors. The paraffin-embedded tumor tissues were cut into 4 μm, which was followed by antigen retrieval and endogenous peroxidase blockade. Next, the tissues were reacted with the primary antibodies Nanog (1:1000) and Oct4 (1:250, both from Abcam) and with the secondary antibody labeled with anti-rabbit HRP. Diaminobenzidine (DAB) was added for color development while hematoxylin for counterstaining. After dehydration, the sections were observed and photographed with a microscope. Image pro plus was included to measure the intensity of the brown (DAB) color (Huang et al. 2019).

### Tumor xenografts in nude mice

Male BALB/c nude mice (4–6 weeks old) were from the experimental animal center of Wuhan University (Hubei, China) and randomly divided into groups (*n* = 25 in each group). P-CSCs and A-CSCs were incubated with 100 μg EVs or PBS (Xu et al. 2019). Then, the cell suspension (1 × 10^7^ cells/mL, 100 μL) was subcutaneously injected into nude mice. Tumor volume: 1/2 × L^2^ × W (L: length, W: width). The tumor growth was observed every 7 days. All nude mice were euthanized on day 35, and the tumor volume and weight were measured.

### Dual luciferase reporter gene assay

The luciferase reporter vector containing the KLF3 3′-UTR sequence was constructed by pmirGLO vector (Promega, WI, USA). pmirGLO-KLF3-wild type (Wt) or pmirGLO-KLF3-mutant (Mut) (500 ng) was co-transfected with miR-21-5p-mimic or NC-mimic into P-CSCs and A-CSCs in 24-well plates by Lipofectamine 3000 (Invitrogen). Reacted for 48 h, the activities of firefly luciferase and Renilla luciferase were measured by a dual luciferase reporter gene detection system (Promega). Relative luciferase activity = firefly luciferase activity/Renilla luciferase activity.

### Statistical analysis

SPSS13.0 statistical software (SPSS Inc., IL, USA) was indicated for data analysis. The data were expressed as mean ± standard deviation. The disparities between two groups were evaluated by independent sample *t* test while those among multiple groups by one-way analysis of variance (ANOVA), followed by Tukey’s multiple comparisons test. Statistically significance was achieved by *P* < 0.05.

## Results

### Isolation and identification of EVs: miR-21-5p is upregulated in M2 macrophage-derived EVs

**Fig. 1:**
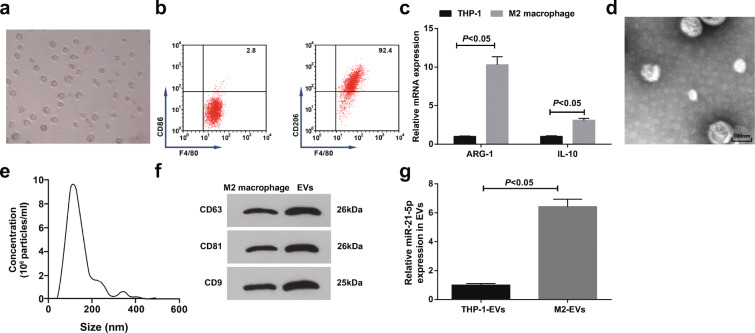
Isolation and identification of EVs; miR-21-5p is upregulated in M2 macrophage-derived EVs. **a** Microscopic observation of M2 macrophages. **b** Flow cytometry detection of F4/80, CD86, and CD206 expression (F4/80 was a pan-macrophage marker). **c** RT-qPCR detection of ARG-1 and IL-10 expression. **d** TEM observation of EVs. **e** Particle size of EVs. **f** Western blot detection of CD63, CD81, and CD9 expression. **g** RT-qPCR detection of miR-21-5p expression in M2 macrophage-derived EVs. *N* = 3; The data were expressed as mean ± standard deviation. The disparities between two groups were evaluated by independent sample *t* test

Infiltration of M2 macrophages is closely connected with the inferior prognosis of PaCa (Michelakos et al. 2020), and M2 macrophage-derived EVs can promote the functions of PaCa cells (Yin et al. 2020). In the experiment, THP-1 cells were induced to polarize into M2 macrophages, which were elliptical and grew aggregately (Fig. 1a). F4/80 was a macrophage phenotype, and CD206 was a unique phenotype of M2 macrophages while CD86 of M1 macrophages. Flow cytometry revealed that CD206/F4/80 was positively expressed (92.4%) while CD86/F4/80 was negatively expressed (2.8%) on the induced macrophages (Fig. 1b). Increased ARG-1 and IL-10 expression have been suggested in polarized M2 macrophages (Jiang et al. 2020; Wang et al. 2020a). Considering that, ARG-1 and IL-10 levels were detected by RT-qPCR and the results indicated that the polarized M2 macrophages showed with upregulated ARG-1 and IL-10 mRNA expression (Fig. 1c), evidencing that M2 macrophages were successfully induced.

Observed by the TEM, M2 macrophage-derived EVs were spherical or elliptical vesicles with intact membranes in a similar shape (Fig. 1d). NTA identified that the average size of EVs was about 120 nm (Fig. 1e). Moreover, CD63, CD81, and CD9 expression in EVs were higher than those in M2 macrophages (Fig. 1f), verifying that M2 macrophage-derived EVs were successfully obtained.

Higher miR-21-5p level in the blood is the critical factor for the poor prognosis of PaCa patients (Qu et al. 2017; Karasek et al. 2018; Vila-Navarro et al. 2019). RT-qPCR revealed the highly expressed miR-21-5p in M2 macrophage-derived EVs versus to THP-1 cell-derived EVs (Fig. 1g), indicating that the upregulated miR-21-5p in M2 macrophage-derived EVs may be tied up with PaCa progression.

### M2 macrophage-derived EVs promote PaCa stem cell differentiation and activity

**Fig. 2:**
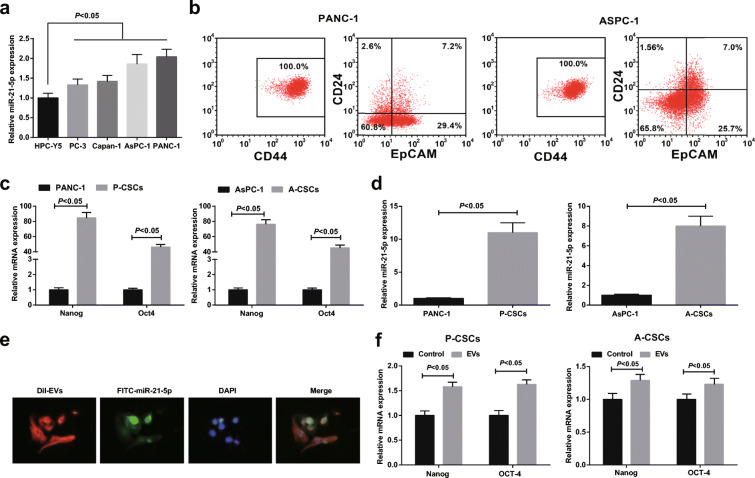
M2 macrophage-derived EVs promote PaCa stem cell differentiation and activity. **a** RT-qPCR detection of miR-21-5p expression in cell lines. **b** Flow cytometry of CD24^+^CD44^+^EpCAM^+^ cell sorting. **c** RT-qPCR detection of Nanog and Oct4 expression. **d** RT-qPCR detection of miR-21-5p expression in cell lines. **e** Internalization of FITC-miR-21-5p-contained EVs by PaCa stem cells. **f** RT-qPCR detection of Nanog and Oct4 expression after EV treatment; *N* = 3; The data were expressed as mean ± standard deviation. The disparities between two groups were evaluated by independent sample *t* test while those among multiple groups by one-way ANOVA, followed by Tukey’s multiple comparisons test

Tumor stem cells are the original cause of tumor progression, and PaCa stem cells have also been proved to be closely related to the poor prognosis of PaCa patients (Rasheed et al. 2010; Nguyen et al. 2017). Therefore, this article was focused on the impacts on PaCa stem cell functions. miR-21-5p expression in PaCa cell lines PC-3, Capan-1, AsPC-1, and PANC-1 and human normal pancreatic cells HPC-Y5 was detected by RT-qPCR. Given the fact that PANC-1 and AsPC-1 presented miR-21-5p at a dramatically high level (Fig. 2a), they were selected for the proceeding experiments. PaCa stem cell marker CD24 (Eng et al. 2016) was detected and CD24 and EpCAM were sorted by flow cytometry. It was revealed that PANC-1 cells expressed CD44 (100%). By detecting CD24 and EpCAM via flow cytometry, CD24^+^CD44^+^EpCAM^+^ cells accounted for 7.2% in PANC-1 cells while 7.0% in AsPC-1 cells (Fig. 2b). CD24^+^CD44^+^EpCAM^+^ cells obtained from AsPC-1 and PANC-1 cells were P-CSCs and A-CSCs, respectively. Nanog and Oct4 are stem cell markers (Wu et al. 2019; Li et al. 2020; Dingle et al. 2021). By RT-qPCR detection of Nanog, Oct4, and miR-21-5p, it was disclosed that they were all upregulated in P-CSCs and A-CSCs versus to PANC-1 and AsPC-1 cells (Fig. 2c, d).

**Fig. 3:**
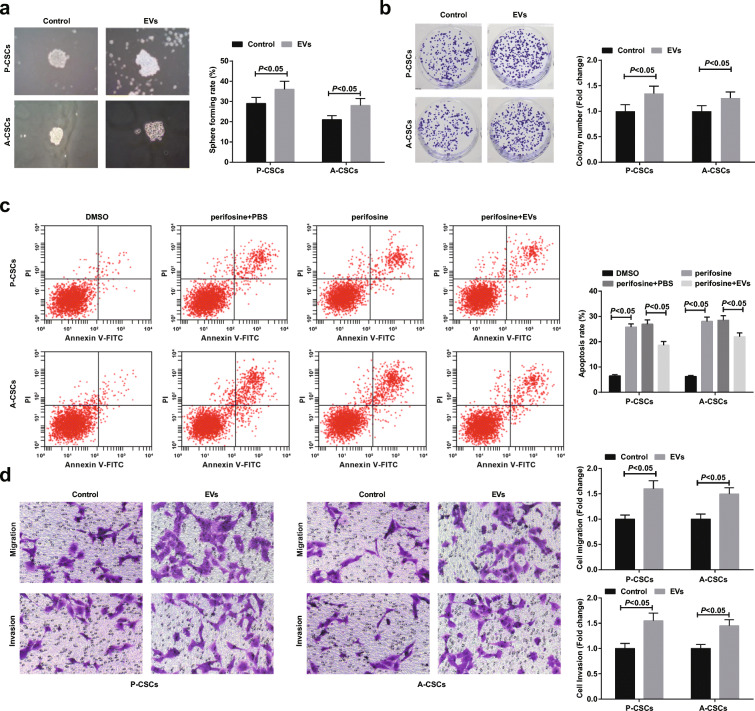
M2 macrophage-derived EVs promote PaCa stem cell differentiation and activity. **a** Tumor sphere formation assay of cell sphere-forming ability, the control group was treated with equal volume of PBS, **b** colony formation assay of cell colony-forming ability, **c** flow cytometry detection of cell apoptosis, and **d** Transwell assay detection of cell migration and invasion. *N* = 3; The data were expressed as mean ± standard deviation. The disparities between two groups were evaluated by independent sample *t* test while those among multiple groups by one-way ANOVA, followed by Tukey’s multiple comparisons test

The effects of M2 macrophage-derived EVs on PaCa stem cells were explored. FITC-miR-21-5p (green) was electroporated to M2 macrophages, from which EVs were extracted, added with Dil (red), and incubated with PaCa cells for 48 h. FITC and Dil were observed in pancreatic cancer stem cells. Co-localization of FITC and Dil in PaCa cells indicated that EVs containing FITC-miR-21-5p were internalized by PaCa cells (Fig. 2e). After EV uptake, Nanog and Oct4 expression were elevated in P-CSCs and A-CSCs (Fig. 2f). Tumor sphere formation, colony formation, and Transwell assays revealed that M2 macrophage-derived EVs enhanced sphere-forming, colony-forming, invasion, and migration abilities of P-CSCs and A-CSCs (Fig. 3a–c). Since perifosine as an Akt inhibitor can induce apoptosis of PaCa cells (Zhou et al. 2020), the anti-apoptotic ability of stem cells after perifosine treatment was measured by flow cytometry. The outcome suggested that M2 macrophage-derived EVs lowered the apoptosis rate of A-CSCs and A-CSCs (Fig. 3d).

### Downregulated miR-21-5p in M2 macrophage-derived EVs suppresses PaCa stem cell differentiation and activity

**Fig. 4:**
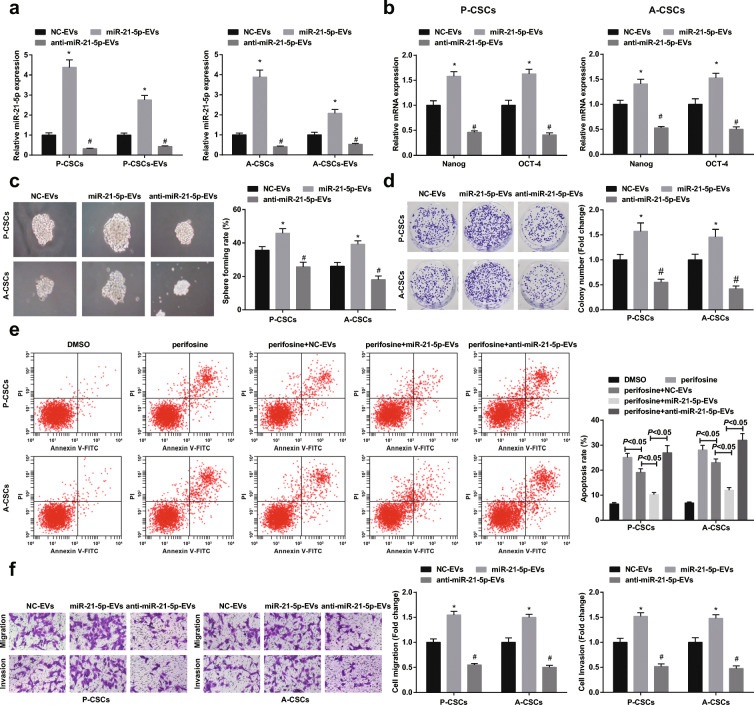
Downregulated miR-21-5p in M2 macrophage-derived EVs suppresses PaCa stem cell differentiation and activity. **a** RT-qPCR detection of miR-21-5p expression after P-CSCs and A-CSCs co-culturing with EVs, **b** RT-qPCR detection Nanog and Oct4 expression, **c** tumor sphere formation assay of cell sphere-forming ability, **d** colony formation assay of cell colony-forming ability, **e** flow cytometry detection of cell apoptosis, and **f** Transwell assay detection of cell migration and invasion. *P < 0.05 compared with the NC-EV group; #*P* < 0.05 compared with the miR-21-5p-EV group. *N* = 3; The data were expressed as mean ± standard deviation. The disparities among multiple groups were evaluated by one-way ANOVA, followed by Tukey’s multiple comparisons test

To explain how miR-21-5p delivered by M2 macrophage-derived EVs performed on PaCa cells, M2 macrophages were stably transfected with downregulated or upregulated miR-21-5p lentivirus and then miR-21-5p expression was demonstrated to downregulate or upregulate in RT-qPCR detection (Fig. 4a). Next, through co-culture with M2 macrophage-derived EVs, P-CSCs and A-CSCs were characterized by decreased or increased miR-21-5p in P-CSCs and A-CSCs. Except for inhibited or promoted Nanog and Oct4 expression (Fig. 4b), miR-21-5p downregulation or upregulation also functioned to impair or enhance sphere-forming, colony-forming, anti-apoptotic, invasion, and migration abilities of P-CSCs and A-CSCs (Fig. 4c–f).

### Downregulated miR-21-5p in M2 macrophage-derived EVs disrupts tumorigenesis of PaCa in mice

**Fig. 5:**
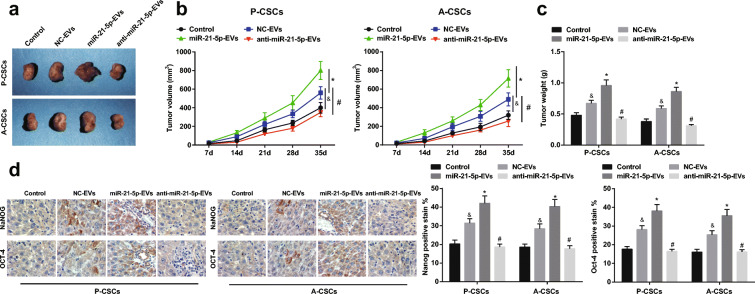
Downregulated miR-21-5p in M2 macrophage-derived EVs disrupts tumorigenesis of PaCa in mice. **a** Subcutaneous xenografted tumors in nude mice, **b** tumor volume growth curve, **c** tumor weight, and **d** immunohistochemistry of Nanog and Oct4 expression. &*P* < 0.05 compared with the control group; *P < 0.05 compared with the NC-EV group; #*P* < 0.05 compared with the miR-21-5p-EV group. *N* = 5; The data were expressed as mean ± standard deviation. The disparities among multiple groups were evaluated by one-way ANOVA, followed by Tukey’s multiple comparisons test

To figure out the impacts of miR-21-5p on PaCa tumorigenesis in vivo, P-CSCs and A-CSCs incubated with M2 macrophage-derived EVs were injected into nude mice. Tumor growth was evaluated by measurements of tumor volume and weight. Downregulated miR-21-5p in M2 macrophage-derived EVs restrained tumor volume and weight while upregulated miR-21-5p in M2 macrophage-derived EVs functioned oppositely (Fig. 5a–c). Moreover, immunohistochemistry of tumors highlighted that downregulated exosomal miR-21-5p reduced Nanog and Oct4 expression while overexpressed exosomal miR-21-5p raised Nanog and Oct4 expression (Fig. 5d).

### miR-21-5p targets KLF3

**Fig. 6:**
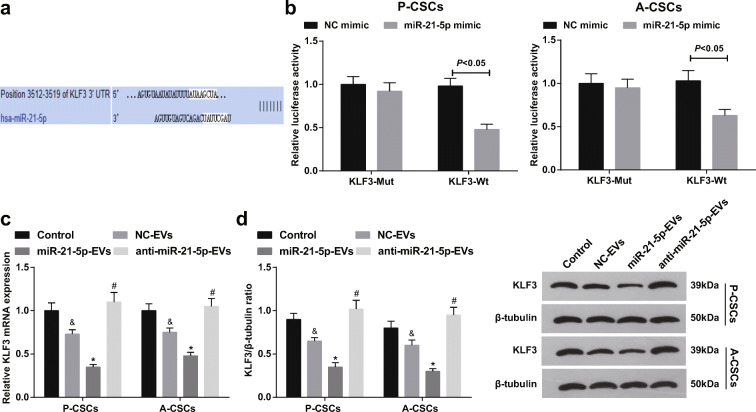
miR-21-5p targets KLF3. a TargetScan prediction of miR-21-5p binding site with KLF3, **b** dual luciferase reporter gene assay verification of the regulatory relationship between miR-21-5p and KLF3 in P-CSCs and A-CSCs cells, **c** RT-qPCR detection of KLF3 mRNA expression in P-CSCs and A-CSCs after EV treatment, and **d** Western blot detection of KLF3 protein expression in P-CSCs and A-CSCs after EV treatment. &*P* < 0.05 compared with the control group; *P < 0.05 compared with the NC-EV group; #*P* < 0.05 compared with the miR-21-5p-EV group. *N* = 3; The data were expressed as mean ± standard deviation. The disparities between two groups were evaluated by independent sample *t* test while those among multiple groups by one-way ANOVA, followed by Tukey’s multiple comparisons test

KLF3 acts as a tumor suppressor gene in cancers and suppresses stemness-related genes (Sun et al. 2019a; Liu et al. 2020a). Based on the predicted binding sites between miR-21-5p and KLF3 through TargetScan (Fig. 6a), dual luciferase reporter gene assay further conformed that miR-21-5p-mimic undermined the luciferase activity of KLF3-Wt in P-CSCs and A-CSCs (Fig. 6b), determining that miR-21-5p interacted with KLF3. Also, upregulating or downregulating miR-21-5p in M2 macrophage-derived EVs reduced or heightened KLF3 expression in P-CSCs and A-CSCs (Fig. 6c, d).

### Restoring KLF3 inhibits PaCa stem cell differentiation and activity

**Fig. 7:**
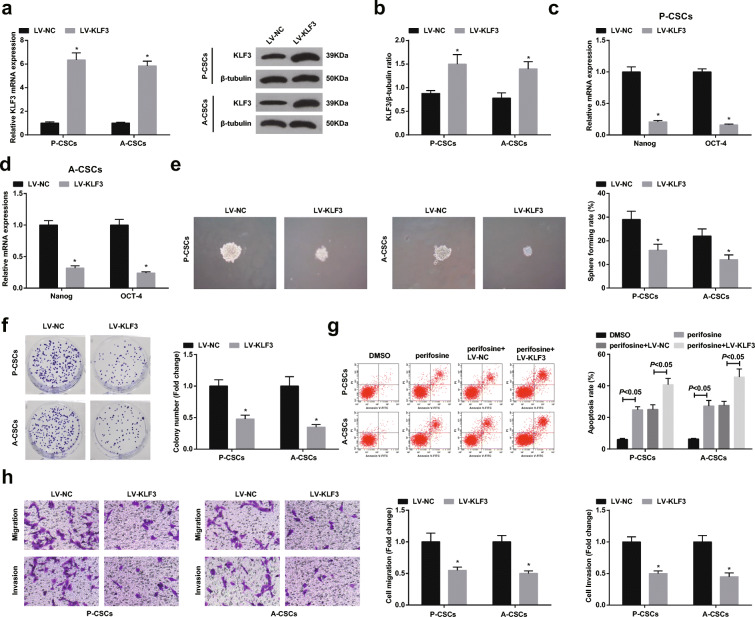
Restoring KLF3 inhibits PaCa stem cell differentiation and activity. **a** RT-qPCR detection of KLF3 expression in P-CSCs and A-CSCs, **b** Western blot detection of KLF3 protein expression in P-CSCs and A-CSCs, **c**, **d** RT-qPCR detection of Nanog and Oct4 expression, **e** tumor sphere formation assay of cell sphere-forming ability, **f** Colony formation assay of cell colony-forming ability, **g** flow cytometry detection of cell apoptosis, and **h** Transwell assay detection of cell migration and invasion. *P < 0.05 compared with the LV-NC group; &*P* < 0.05 compared with the LV-KLF3 group; #*P* < 0.05 compared with the LV-KLF3 + NC-EV group. *N* = 3; The data were expressed as mean ± standard deviation. The disparities among multiple groups were evaluated by one-way ANOVA, followed by Tukey’s multiple comparisons test

Then, we explored whether KLF3 mediated miR-21-5p to affect PaCa stem cell differentiation and activity in P-CSCs and A-CSCs. P-CSCs and A-CSCs were transfected with overexpressed KLF3, in which KLF3 was upregulated (Fig. 7a, b). Upon KLF3 restoration, Nanog and Oct4 expression were suppressed (Fig. 7c, d), and the capacities of P-CSCs and A-CSCs to form spheres and colonies, invade and migrate, and resist apoptosis were disrupted (Fig. 7e–h). However, augmenting miR-21-5p mitigated the effects of restored KLF3 on P-CSCs and A-CSCs.

## Discussion

PaCa is a complex disease that causes tumor fibrosis and aggressiveness (Khan et al. 2019). By various assays, we have noticed and verified that M2 macrophage-derived EVs participate in PaCa progression, especially in PaCa stem cell differentiation and activity. Firstly, M2 macrophage-derived EVs are proved to promote the sphere-forming, colony-forming, invasion, migration, and anti-apoptosis capacities of PaCa stem cells. Next, miR-21a-5p is found to upregulate in M2 macrophage-derived EVs and miR-21a-5p downregulation in M2 macrophage-derived EVs inhibits PaCa stem cell differentiation and activities. It is also figured out that upregulated miR-21-5p directly targets KLF3, thereafter to regulate PaCa stem cells.

M2 macrophage-derived exosomes are validated to aggrandize the proliferative, migratory, invasive, and anti-apoptosis capacities of PaCa cells (Yin et al. 2020). In addition to that, M2 macrophage-derived exosomes top on the ideal position for promoting PDAC cells to migrate and invade, and form tumors (Yin et al. 2019). In hepatocellular carcinoma, M2 macrophage-derived exosomes provide a niche for cell migration mediated by tumor-associated macrophages and mediate the transportation of CD11b/CD18 to activate matrix metalloproteinase-9 signaling pathway, thereby stimulating cell metastasis (Wu et al. 2020). Moreover, in epithelial ovarian cancer, M2 macrophage-secreted exosomes induce faster proliferation and increased G_1_/S cell cycle entry (Li and Tang 2020). Experimentally, M2 macrophage-derived exosomes establish the base for gastric cancer cell migration through mediating the delivery of ApoE protein from tumor-associated macrophages to the tumor cells (Zheng et al. 2018). Supplemented to this work, M2 macrophage-secreted exosomes treated with miR-21-5p inhibitor, exhibit reduced miR-21-5p expression, and depleted miR-21-5p in M2 macrophage-secreted exosomes impairs the motility, invasion, and migration of CRC cells (Lan et al. 2019). Mechanistically, miR-21 is evidenced to upregulate in M2 macrophage-derived exosomes and can transfer from M2 macrophages to gastric cancer cells, thereafter to disrupt cell apoptosis (Zheng et al. 2017).

miR-21-5p is commonly defined as an oncogene in various solid tumors. In lung adenocarcinoma, upregulated miR-21-5p is tended to facilitate the aggressive activities of cancer cells while silenced miR-21-5p exerts conversely (Wang et al. 2020d). Also, during the progression of non-small cell lung cancer, elevated miR-21-5p substantially contributes to the proliferative and anti-apoptotic properties of cancer cells while reduced miR-21-5p works out with the opposite results (Yang et al. 2020). In a similar way, the manifestation of miR-21-5p in breast cancer goes toward an elevation and miR-21-5p inhibition destructs the biological functions of cancer cells (Xie et al. 2019). In the progression of PaCa, miR-21 is demonstrated to up-regulate in pancreatic adenocarcinoma (Lu et al. 2020) while miR-21-5p shows the same trend in pancreatic neoplasia (Karasek et al. 2018; Melisi et al. 2019; Vila-Navarro et al. 2019). The suppressed miR-21, packaged in tumor-penetrating nanoparticles, is transferred into the tumor site, attaining the achievement of delaying the formation of PDAC tumors (Gilles et al. 2019). Silencing miR-21 restrains the tumorigenic activities of cancer cells and miR-21 suppression in an early stage blocks the tumor progression of PDAC (Chu et al. 2020). Intriguingly, depleting miR-21 activates its potency to impede the PaCa cell progression and slow down tumorigenesis in vivo (Sun et al. 2019b).

KLF3 the target gene of miR-21 (Zhai et al. 2019) is suggested to be an anti-tumor actor in tumors. Considering that, it is speculated that miR-21-5p may mediate KLF3 to participate in the progression of PaCa stem cells. In lung cancer, inhibited KLF3 is documented to precipitate EMT and metastasis (Sun et al. 2019a). KLF3 expression is restrained in CRC, which accelerates cell proliferation and migration (Lv et al. 2020). Besides, a decrease is manifested in KLF3 expression in esophageal squamous cell carcinoma and further depletion of KLF3 augments the migration and invasion of cancer cells (Liu et al. 2020a). As evidenced by a former study, KLF3 can be utilized in predicting the overall survival because of its reduced expression, and elimination of KLF3 triggers the aggressive phenotype of CRC (Wang et al. 2017).

All in all, this work has made it explanatory that downregulated miR-21-5p in M2 macrophage-secreted EVs raises KLF3 expression to destroy the pre-malignant activities of PaCa stem cells. Limitations have been recognized in the research scale. Hence, a plenty of researches are still at wanting to explore the mechanism of PaCa stemness.

## Data Availability

Not applicable
